# Why does the recurrent implantation failure literature need to be rewritten?

**DOI:** 10.1093/hropen/hoaf035

**Published:** 2025-06-18

**Authors:** Baris Ata

**Affiliations:** Department of Obstetrics and Gynecology, Koç University School of Medicine, Istanbul, Turkey; ART Fertility Clinics, Dubai, United Arab Emirates

The answer to the question posed in the title is straightforward: the vast majority of studies on recurrent implantation failure (RIF) appear to be irrelevant. Most included patients had simply experienced an arbitrarily defined number of embryo transfer failures. Often, the cumulative number of embryos transferred before a study participant qualified as having RIF conferred a very low cumulative probability of implantation or live birth—even if the only issue was embryonic aneuploidy. In many cases, aneuploidy in the transferred embryos could likely explain the failures, even if no other underlying pathology was present.

A well-conducted systematic review in the current issue of *Human Reproduction Open* offers irrefutable evidence of this situation by providing a snapshot of the RIF literature (Lu *et al.*, 2025). Lu and colleagues cataloged the definitions of RIF used in 748 studies identified through a systematic search using the terms ‘recurrent implantation failure’ or ‘repeated implantation failure’. Astonishingly, they found **503 unique definitions**. Moreover, 11.3% of the studies (86) used multiple definitions, and 9.8% (73) did not define RIF at all. Furthermore, 34.8% of studies failed to report the number of embryos transferred, and 59.9% required only four or fewer embryos to have failed before labeling a patient as having RIF. Despite substantial differences in implantation rates between cleavage-stage and blastocyst-stage embryos, 81.2% of the studies did not report the developmental stage of the failed embryos. Among the 18.8% that did, nearly half (47.4%) of the cleavage-stage studies required only 4–5 embryos to have failed, and all blastocyst-based studies required only 2–4 failures to meet the RIF threshold.

The current paradigm for defining RIF is evolving toward basing the diagnosis on the failure to achieve implantation after a number of embryo transfers sufficient to yield a **predefined cumulative chance of pregnancy**. Although this threshold is still arbitrary, as Lu *et al.* (2025) emphasize, it offers a more rational benchmark. We previously proposed a stringent threshold of 95% and reported that even in younger women, an average of seven unscreened blastocysts is needed to achieve a 95% cumulative implantation probability—assuming aneuploidy is the sole issue (Ata *et al.*, 2021). The required number would be even higher for cleavage-stage embryos.

Lu *et al.* (2025) estimated anticipated cumulative pregnancy probabilities based on patient age and number of embryos transferred prior to enrolment in the original RIF studies. Remarkably, only ∼1% of studies reached a 95% cumulative probability. The ESHRE Good Practice Recommendations suggest a more lenient 60% threshold (ESHRE Working Group on Recurrent Implantation Failure *et al.*, 2023). Even by this standard, only 6.5% of the studies included women who met the criterion. In alternative models developed by Lu *et al.* (2025), the proportion of studies meeting the ESHRE threshold ranged from 2.1% to 34.5%. Notably, even when all participants were assumed to be under the age of 35 in the studies that reported number of embryos transferred, 34.9% of the studies still failed to meet the lenient ESHRE criterion.

It appears that many participants in RIF studies may have experienced implantation failure due to randomly occurring aneuploidy rather than unrecognized embryonic or uterine pathologies. Including such patients alongside those with genuine pathology carries significant implications for both clinical practice and research.

First and foremost, random positive findings from such heterogeneous cohorts risk leading patients worldwide to undergo unnecessary tests and interventions. Equally concerning, diluting genuine RIF cases makes it difficult to identify and validate rare but real causes of implantation failure. Given that, in the absence of known pathology, each of five consecutive euploid blastocysts has a comparable live birth rate regardless of prior failures—and that cumulative live birth probability exceeds 98%—any undiagnosed uterine pathology must affect fewer than 2% of patients (Gill *et al.*, 2024). Finding such rare causes in a mixed cohort is, figuratively speaking, like searching for a needle in a haystack.

Lenient RIF definitions therefore risk rendering future studies underpowered, leading to false-negative results and ultimately **hindering** progress in implantation failure research. The systematic review by Lu *et al.* (2025) is both timely and necessary. It clearly highlights the problem using objective data from the literature and calls for a rewriting of the field using more stringent and consistent RIF criteria. While it sounds difficult to complete recruitment for a study with stringent criteria, this is not necessarily bad news. Maybe RIF is much less prevalent than formerly thought or does not even exist ((The writing group) for the participants to the 2022 Lugano RIF Workshop *et al.*, 2023; Gill *et al.*, 2024).

## Author’s roles

B.A. conceived and wrote the manuscript.
